# The power and promise of interdisciplinary international research networks to advance movement ecology

**DOI:** 10.1186/s40462-023-00428-8

**Published:** 2023-10-23

**Authors:** Ivan Jarić, Robert J. Lennox, Marie Prchalová, Christopher T. Monk, Milan Říha, Ran Nathan, Robert Arlinghaus

**Affiliations:** 1 Université Paris-Saclay, CNRS, AgroParisTech, Ecologie Systématique Evolution, Gif sur Yvette, France; 2 Biology Centre of the Czech Academy of Sciences, Institute of Hydrobiology, České Budějovice, Czech Republic; 3 Ocean Tracking Network, Department of Biology, Dalhousie University, Halifax, Canada; 4 Laboratory for Freshwater Ecology and Inland Fisheries, NORCE Norwegian Research Centre, Bergen, Norway; 5 GEOMAR Helmholtz Centre for Ocean Research, Kiel, Germany; 6 Movement Ecology Lab, A. Silberman Institute of Life Sciences, The Hebrew University of Jerusalem, Edmond J. Safra Campus, Jerusalem, Israel; 7 Minerva Center for Movement Ecology, The Hebrew University of Jerusalem, Jerusalem, Israel; 8 Department of Fish Biology, Fisheries and Aquaculture, Leibniz Institute of Freshwater Ecology and Inland Fisheries, Berlin, Germany; 9 Division of Integrative Fisheries Management, Faculty of Life Sciences and Integrative Research Institute on Transformations of Human-Environment Systems (IRI THESys), Humboldt-Universität zu Berlin, Berlin, Germany

One of the most important tasks of ecology is to understand how animals use space and time. Recent advances in the development of automated telemetry systems have enabled tremendous progress in understanding animal ecology, distribution, and behavior in both terrestrial and aquatic environments [1–3]. The field of biotelemetry has shifted rapidly from data-poor to data-rich field, when new technologies started to provide huge amounts of data about tracked animals, with unprecedented temporal and spatial resolution [3]. However, these new tracking tools also bring many challenges that can be effectively mitigated only through international and interdisciplinary collaborative efforts. Such initiatives represent indispensable platforms for sharing data, ideas, and technical capacity, establishment of common protocols and standards, efforts to address research questions at broader scales, implementation of international and transdisciplinary research projects, and facilitated uptake of obtained knowledge and information to inform governance and policy [4, 5].

Data sharing is one of the major goals of collaborative research networks, because sharing provides a unique opportunity to upscale data to landscape or ecosystem scales, which is simply intractable for individual research groups to accomplish independently, given the costs and logistics of instrumenting multiple areas for replicated research. Archiving data according to FAIR principles [6] and allowing connections to be forged across research groups is essential for ecologists to leverage the power of acoustic tracking against the high direct and indirect costs of project implementation.

There is a growing number of international networks for collecting and sharing telemetry data that operate around the world, including the Ocean Tracking Network (OTN), Integrated Marine Observing System (IMOS), European Tracking Network (ETN), Great Lakes Acoustic Observation System (GLATOS), Acoustic Tracking Array Platform (ATAP), and Lake Fish Telemetry Group (LFTG) [7–11].

The LFTG was established to advance topics in aquatic ecology using shared data sets, data systems, and ecosystem thinking. We present here a brief overview of the LFTG, as an example of an international and interdisciplinary network, with its positive features, challenges, and lessons learned. We further provide an overview of the thematic series ‘Advancing Movement Ecology Through Freshwater Fish Tracking’, which has been initiated through this network, and discuss key future challenges regarding data sharing within and among such networks.

## Lake fish telemetry group (LFTG)

Fine-scale telemetry systems tend to be located in freshwater systems, mainly lakes, due to the need for enclosed systems to perform long-term tracking without losing tracked fish through emigration [1]. Lakes also allow dense receiver networks to conduct positioning, which is less tractable in saltwater environments where acoustic signals are attenuated more quickly.

The LFTG originated from the desire to build collaborative projects from groups working with fine-scale acoustic telemetry systems in freshwater lentic environments. The concept emerged during a lunch time meeting in Berlin among Ivan Jarić, Milan Říha, Robert Arlinghaus and Christopher Monk, during which they intended to organize a joint project investigating the winter ecology of freshwater fish species in their respective acoustic telemetry datasets. From those discussions a concept emerged, to bring together additional partners with similar study systems, to expand the datasets further. This led to the establishment of LFTG, with a series of annual workshops funded by ALTER-NET and partly supported by the ETN EU COST Action program.

LFTG workshops (organized in 2018, 2019 and 2022) each involved 30–34 participants, mainly research groups from Europe, and beside the groups working on lacustrine telemetry systems, they also involved several groups working in rivers and saltwater systems. The primary goal of the workshops was to develop joint collaborative projects with empirical data, but the workshops were designed to also jointly develop conceptual work, and to share knowledge and experience. Topics covered included winter ecology, circadian rhythms, 3D analysis, high-throughput movement ecology, the importance of lakes, the history of positioning and fine-scale fish tracking, and trends in telemetry in lakes. The workshops have largely been a success, resulting in multiple joint publications [1, 3, 12, 13], as well as this thematic series.

Several workshop design choices contributed to the productivity of the meetings. Firstly, outside experts were invited to each of the meetings, including those working with high resolution biologging systems in terrestrial ecosystems, statisticians, and behavioural ecologists. In this way, new knowledge could be injected into the work conducted at the workshops, beyond the relatively similar training and academic backgrounds of the fish ecologists working with acoustic telemetry. The fact that fine-scale tracking systems are relatively rare was also serendipitously helpful for managing the group size. Secondly, each workshop began with a day of seminars, in which groups could present their research programs, and the latest results or projects. In this way, each group was eased into the workshop and had time to understand where their work could fit in with others. Thirdly, many joint projects were proposed, some of which were successes and many of which fell flat, or require more time to completion; focusing on a larger number of potential projects increased the likelihood that certain subset would become successful.

The initial objective of the LFTG was to combine, scale up and synthesize empirical datasets. However, the output of the LFTG to date have been mainly conceptual papers and reviews. This speaks to the difficulty of jointly performing empirical analyses. In our experience, writing is easily a very collaborative effort, particularly now with shared online text processing tools such as google docs. By contrast, data analysis often landed in the hands of the leader of the joint project. The analysis of fine-scale data can be a cumbersome task even for a single study site, and the joint project can require considerable additional efforts. The leader has to synchronize formats of multiple datasets, solve problems related to diverse study designs, such as inconsistent tracking durations, burst rates, system errors, and fish traits (e.g., size), and ensure greater computation power.

For future similar endeavors, more effort up front to build a shared database, with standard data formats would have reduced major efforts later in starting projects. Moreover, we recommend the use of collaborative online scripting tools (e.g., jupyter notebooks, observablehq) and github repositories to share the effort of analysis and synergize the data analysis knowledge of the participants.

## About the thematic series

The thematic series ‘Advancing Movement Ecology Through Freshwater Fish Tracking’, initiated during the third workshop of the LFTG in May 2022, aimed to showcase the power and potential of animal tracking to address a range of research questions related to the movement ecology of freshwater fish, as well as applied topics related to fisheries management and conservation. The thematic series comprises 15 contributions, with the majority represented by research articles, as well as two reviews and one perspective. Studies were conducted in Europe and North America, reflecting the relatively narrow distribution of freshwater animal tracking efforts [14], and predominantly focused on cyprinid, salmonid, and percid species. They addressed a wide range of research topics, including activity, fine-scale movement, spawning migrations, overwintering behaviour, social networks, distribution, space use, habitat preferences, and effects of abiotic factors.

Brönmark et al. [15] discuss the importance of whole-lake studies and replicated pond systems as an innovative experimental infrastructure, to study animal movement and ecology. They also present their experiences with iPonds as a case study. iPonds, an experimental pond infrastructure in Sweden equipped with an acoustic telemetry system, allow different experimental designs, including observational evaluation of individual behaviours in space and time, sequential addition or removal of experimental treatments, and simultaneous data collection of differently manipulated subsets of individual fish. Authors also discuss key limitations and challenges, and emerging future directions using such systems, such as predator-prey interactions, the ‘landscape of fear’ concept, predator avoidance-foraging trade-offs, individual differences in fear perception, the process of acquiring information on predation threats, physiology, environmental factors, social interactions, and fish-angler interactions.

Two reviews address particular issues from the movement ecology toolbox, the use of accelerometer transmitters and space-use estimators. Lennox et al. [16] review the use of accelerometers in aquatic sciences. The review includes 53 articles, covering a wide range of aquatic species, including fish, reptiles and invertebrates. The main applications of accelerometers so far included analyses of activity and survival, exercise physiology, response to and recovery from stressors, energetics, conservation planning, and behaviour identification. Their analysis reveals an important problem of a lack of standardization among studies, with a wide variation in the sampling frequencies and windows used that may negatively affect the utility of data and the potential for comparisons across studies. Kraft et al. [17] review the use of various estimators of animal space use for acoustic telemetry data. They focus on methods for estimating positions, pseudo-positions, residency and two-dimensional home and occurrence range, such as hull-based methods, density-distribution methods, a network-based approach, and three-dimensional methods, and demonstrate and test their use on samples of thornback ray (*Raja clavata*) and common stingray (*Dasyatis pastinaca*) telemetry data. They also developed a decision framework for selecting optimum methods based on specific research aims and data characteristics, and argue that the process of method selection also needs to consider advantages and drawbacks of each method, type of data, study design, researcher experience, monetary restrictions and processing capacities.

Monk et al. [18] explore social network dynamics in a social fish, common carp (*Cyprinus carpio*), at various time-scales. They examine seasonal and diurnal rhythms, the strength of social attraction, and the presence of persistent characteristic groups over time. The study shows that the social attraction is a key driver for interactions, with social memory of common carp being mostly limited to two weeks. Shoaling behaviour is more pronounced during wintertime, while the frequency of social interactions declines during summertime. The study provides strong indices of the ability of carp to recognize one another, and represents a promising template for future similar studies.

Westrelin et al. [19] explore the overwintering behaviour of the European catfish (*Silurus glanis*) in a shallow lake in South-Eastern France, focusing on the dynamics of their winter aggregations, and the key driving factors. They found that the aggregations are maintained for up to two months during winter, with a strong site fidelity, and they are more stable when temperatures decrease. Larger fish tends to leave aggregations more frequently, while individual differences in aggregation preference reveal potential presence of distinct behavioural types. Beside ecological insights in fish behaviour, the study also provides practical information for population management, particularly for control measures in areas where the species represents an invasive alien species.

Blanchfield et al. [20] assess seasonal drivers of activity, habitat use and diet of lake trout (*Salvelinus namaycush*) in a subarctic lake in Canada. They estimate fish home ranges, movement rates, tail beat activity, depth use, and seasonal nearshore habitat use, coupled with data on seasonal diet and abiotic conditions. Nearshore habitat is shown to be important during the larger part of the year, and its use peaks each spring and fall. The diet reflects seasonal patterns, with feeding on invertebrates in spring and foraging offshore in summer. Their study demonstrates how this mobile top predator establishes littoral-pelagic habitat coupling by linking nearshore and offshore habitats.

Brooks et al. [21] examine the effects of thermal stratification and deoxygenation on walleye (*Sander vitreus*) movement and habitat compression in a hypoxia-prone coastal embayment, the Hamilton Harbour in the Lake Ontario. Thermal stratification and a hypoxic hypolimnion are shown to substantially reduce their movements and space use, and the amount of suitable habitat. However, tracked fish remain in the harbour for most of the year despite poor water conditions, likely because the remaining, more suitable habitat improved in quality. The authors demonstrate that studies focused on the assessment of fish habitat size and behavior need to consider both habitat quality and quantity.

Two studies investigate effects of environmental cues on spawning migrations based on multi-annual telemetry data. Massie et al. [22] examine how hydrological conditions and environmental cues trigger spawning migrations and the number of migrants of common snook (*Centropomus undecimalis*) in the Everglades National Park in Florida. Their study provides strong evidence of the importance of flow patterns on the migratory behaviour. Importantly, the study reveals that the decisions whether and when to migrate are affected by different combinations of factors, with the proportion of migrants mainly driven by the extent of minimum marsh water and fish size, and the migration timing triggered by high river water and its rate of change. They also observed a high frequency of skipped spawning, which likely serves as an adaptive behaviour to mitigate impacts of environmental variability. Šmejkal et al. [23] monitor reproductive aggregations of asp (*Leuciscus aspius*) in a Želivka Reservoir tributary in the Czech Republic in relation to different environmental cues, including water temperature, discharge, temperature, precipitation, atmospheric pressure, wind speed and lunar phase, as well as temporal changes. The study shows that the observed variability is sex-dependent, with reproductive aggregation size being positively affected by water temperature and wind speed, and highest during the period before and after the full moon and at night. It also demonstrates that a wide range of environmental factors need to be considered to adequately capture the complexity of phenological triggering by environmental cues. Beside their value to inform management, both studies also demonstrate the importance of long-term movement data to reveal patterns that cannot be detected with shorter studies.

Several studies focus on applied research questions, bridging the gap between movement ecology and management. Raboin et al. [24] test the use of CO_2_ as a behavioural non-physical deterrent, to prevent the spread of invasive alien fish species. They study behavioural states of the invasive common carp in navigation locks on the Fox River in Wisconsin in the presence of elevated CO_2_, injected and mixed in the water through the forced water circulation. The study provides evidence that both the elevated CO_2_ concentrations in water and forced water circulation are effective in expelling the fish from the navigation lock. Van Leeuwen et al. [25] explore the habitat use of five cyprinid and percid species as a part of a habitat restoration and enhancement project in the Lake Markermeer in Netherlands. Both omnivorous and piscivorous species are shown to move into the newly created habitat in early spring, during spawning, with piscivorous species remaining resident in the area throughout the year. The results confirm the value of the habitat enhancement measures, as well as of the high-resolution tracking to detect changes in fish communities as a result of habitat change. The study by Pease et al. [26] focuses on movement and catchability of stocked rainbow trout (*Oncorhynchus mykiss*) in 15 lakes in western Washington. They examine the contribution to fisheries of triploid, infertile and artificially produced fish, extensively stocked to support recreational fishing and prevent genetic introgression in wild populations. Fish tracking data are used to compare catchability, fine-scale movement, emigration from the lake and natural mortality of triploid and regular diploid fish, and coupled with data from traditional stock assessments. The study confirms the relevance of triploid trout for fisheries, and provides valuable insights into the movement patterns of stocked fish to inform management and help maximize catch rates. Zdasiuk et al. [27] examine the effects of hydrological alterations on movement of the threatened grass pickerel (*Esox americanus vermiculatus*) in an agricultural drain, through a combination of mark-recapture surveys and passive tracking. While there is a lack of clear relationship between movement and habitat characteristics, results indicate the presence of a density-dependent movement, with more mobile fish in areas with a higher abundance, and a high level of individual variation in mobility. The study reveals that grass pickerel are generally stationary, and that their low mobility may not ensure survival during severe environmental conditions that may be encountered in agricultural drainages. Authors argue that effective conservation management will require establishment of movement corridors between populations to mitigate habitat fragmentation.

Two studies also focus on the impacts of hydropower plants on fish migrations. Using the tags instrumented with accelerometer, temperature, and depth sensors, Dahlmo et al. [28] study the importance of lakes for sea trout (*Salmo trutta*) spawning migrations, and the effects of hydropower water discharge on their behaviour. The study reveals that the lake provides an important habitat for sea trout during spawning migrations, and likely realization of spawning within the lake. Lower fish activity observed in the lake compared to the river indicates that lakes may act as a refuge habitat that can be used by fish to conserve energy and prepare for spawning. The study did not detect significant impacts of hydropower water discharge on sea trout behaviour in the lake. Mawer et al. [29] study upstream migrations of two fish species at the hydropower plant on the River Iller in Germany, and their habitat preferences as they approach the fish pass. Step selection functions are used to quantify individual and species-specific differences in the choice of hydraulic parameters. They observe that barbel (*Barbus barbus*) prefers faster flows, deeper water, and higher velocity gradients, while grayling (*Thymallus thymallus*) shows strong individual variation and thus lacks consistent general trends.

## Future of telemetry data sharing

The different contributions in this thematic series effectively showcase the potential of freshwater animal tracking to address a range of theoretical and applied research questions. Additionally, some of the studies [15, 26] highlight the importance of the use of replicated tracking systems, which will require considerable improvement of international collaborations and data sharing, and overcoming present obstacles to such initiatives. Barriers to sharing data in the field of telemetry have been described by Nguyen et al. [5], but an updated perspective is needed to track progress in the field, as sharing becomes more conventional and there is a top-down pressure from funding bodies to make data available. Each network has a specific data policy that protects data according to the stipulations of the researcher and data are not openly available without consent of the researcher. Importantly, these databases are all designed to handle detection data and not position data. New infrastructural solutions are needed to handle position data, to facilitate high-resolution synthesis activities – however, this will require solutions including whether to archive raw data, calculated positions, or both. Integrating positioning data into major databases will introduce large volumes of new data but will open new opportunities for replicated synthesis studies of animal movements.

## Data Availability

Not applicable (there were no data or materials collected or generated within this study).
